# Glucocorticoids activate TGF-β induced PAI-1 and CTGF expression in rat hepatocytes

**DOI:** 10.1186/1476-5926-6-5

**Published:** 2007-05-02

**Authors:** Lucia Wickert, Nicolas Chatain, Karin Kruschinsky, Axel M Gressner

**Affiliations:** 1 Institute of Clinical Chemistry and Pathobiochemistry, University Hospital RWTH Aachen, Pauwelsstr. 30, D-52074 Aachen, Germany

## Abstract

**Background:**

In addition to the activation of hepatic stellate cells TGF-β govern apoptosis and growth control of hepatocytes in liver injury. In non-parenchymal cells, TGF-β induces plasminogen activator inhibitor 1 (PAI-1) and connective tissue growth factor (CTGF) expression, which are involved in extra cellular matrix formation. Both genes were also regulated by glucocorticoids, which in certain cases showed antagonistic effects to the TGF-β-Smad 3 pathway. The purpose of our work was to investigate the influence of TGF-β and dexamethasone on PAI-1 and CTGF expression and secretion in primary hepatocytes.

**Results:**

By examining PAI-1 and CTGF mRNA and protein expression in cell lysates and cell-conditioned media under the influence of TGF-β and dexamethasone, we analysed signalling pathways controlling their expression. TGF-β and dexamethasone significantly co-induce PAI-1 and CTGF protein expression. On the other hand, we showed that TGF-β diminished a glucocorticoid receptor dependent luciferase reporter signal in Hep-G2. Inhibition of Erk downstream activation decreased TGF-β induced CTGF and PAI-1 expression to a basal level. PAI-1 was directly secreted by hepatocytes, whereas secretion of CTGF was retarded.

**Conclusion:**

The data provide evidence that beside the TGF-β-Smad 3 pathway CTGF and PAI-1 expression is additionally dependent on Erk activity in hepatocytes giving new insights into regulation of the profibrogenic proteins.

## Background

Hepatocytes fulfil several complex functions, which are regulated by a fine network of transcriptional regulators. Particularly glucocorticoids govern crucial functions in hepatocytes such as glucose metabolism, cell proliferation and differentiation. Once bound to their cytoplasmic receptors (GR) the glucocorticoid-receptor-complex is translocated to the nucleus and binds after dimerization to a cis-active response element to regulate target genes [1,2]. Additionally, ligand-bound GR can interact DNA-independently with transcription factors such as AP-1 or NFκB, resulting in anti-inflammatory effects [3]. These properties were pharmaceutically utilised [4]. In liver injury, the fibrogenetic master cytokine TGF-β activates hepatic stellate cells (HSC) and induces them to produce extra cellular matrix (ECM). Further, TGF-β severely impaired viability and function of hepatocytes [5]. For this, active TGF-β binds and phosphorylates transmembrane receptors with a serine-threonine kinase activity, which in turn propagates the signal via Smads to the nucleus in order to bind corresponding promoter elements [6-8].

PAI-1, a member of the serpin superfamily, is regulated by many different factors among which TGF-β and corticosteroids are important one [9,10]. In HSC, TGF-β induction of PAI-1 leads to an inhibition of protease-dependent fibrinolytic activity and ECM accumulation [11]. CTGF, a CCN family protein and also a TGF-β target gene, is described as a down-stream modulator of profibrotic TGF-β effects, *e.g*. in activating HSC and promoting the deposition of ECM [12-16]. CTGF inhibition is reported to suppress liver fibrosis in rats by inhibiting HSC-activation [17]. Both PAI-1 and CTGF are inducible by glucocorticoids [18-20]. Previous studies showed that glucocorticoids decrease TGF-β-sensitive reporter signalling in primary HSC as well as in CFSC (cirrhotic fat storing cells) [21,22], but little is known about the effect of glucocorticoids on endogenous TGF-β target genes in hepatocytes. Aim of the present study was to characterize the molecular effects of glucocorticoids on PAI-1 and CTGF and to ascertain the impact of glucocorticoid-TGF-β interaction. Therefore, we analysed mRNA and protein expression of PAI-1 and CTGF by a combined stimulation with dexamethasone and TGF-β in primary rat hepatocytes and found that both proteins are co-induced by TGF-β and dexamethasone. CTGF as well as PAI-1 expression control is accomplished by the use of several pathways and our data indicate that a complex signalling network regulate the fine tuning of these proteins in hepatocytes.

## Results

### PAI-1 and CTGF expression after TGF-β dexamethasone costimulation

**Figure 1:**
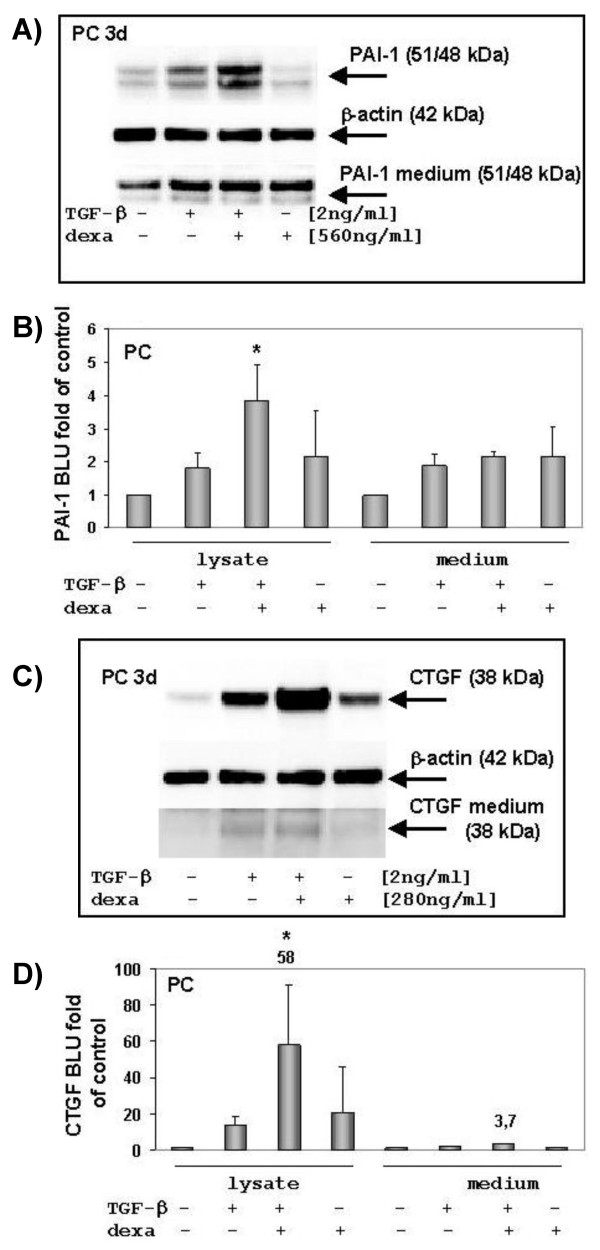
**Western blot analysis of PAI-1 and CTGF expression in hepatocytes**. Cells were incubated 6 hours with TGF-β, dexamethasone or both. Afterwards the hepatocytes were harvested and lysates (40 μg) have been subjected to Western blotting and analyzed for PAI-1 and CTGF expression. (A) PAI-1 was significantly induced after co-stimulation with TGF-β and dexamethasone, indicating a synergistic effect in hepatocytes. β-actin was assayed as loading control. (B) Densitometric analysis of PAI-1 expression in hepatocytes and its release into medium. Three independent western blots were analysed; control *versus *TGF-β and dexamethasone co-treated cells * *p *< 0.05; BLU (Boehringer light units). (C) A co-treatment with TGF-β and dexamethasone strongly induce CTGF in hepatocytes (PC, day three after plating). A low amount of CTGF could be detected in the supernatant of TGF-β stimulated cells. The same blots were analysed for β-actin as loading control. (D) Densitometric analysis of three independent experiments. CTGF is induced about 50-fold after a TGF-β-dexamethasone co-stimulation (control *versus *TGF-β and dexamethasone co-treated cells * *p *< 0.005), but it is hardly secreted.

**Figure 2:**
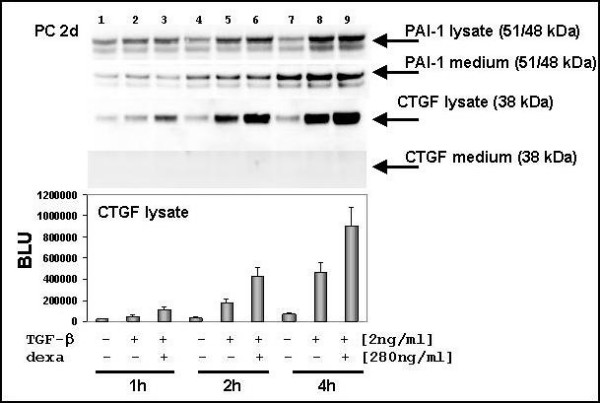
**Differential secretion of PAI-1 and CTGF in hepatocytes**. Western blot analysis of medium samples, which were collected just before cell lysate was prepared. Hepatocytes were seeded in 90 mm^2 ^petri dishes with a density of 5–6 × 10^6 ^cells/well. Before cytokine and hormone stimulation cells medium was changed (5 ml DMEM without FCS and without phenol red). Thereafter, cells were treated and harvested by and by as indicated and 40 μg protein was applied to each lane. After stimulation PAI-1 protein increased during incubation time (1 to 4 h) and was rapidly secreted. CTGF was up-regulated and strong expressed in hepatocytes after co-stimulation with TGF-β and dexamethasone. In a time-frame of four hours no CTGF signal could be detected. The time-dependent induction of CTGF was analysed densitometrically.

Western blot analysis showed an up-regulation of PAI-1 after TGF-β incubation and a strong enhancement six hours after a TGF-β-dexamethasone costimulation of hepatocytes (Fig. 1A, 1B). Whereas no CTGF is detectable in freshly isolated hepatocytes it is up-regulated by degrees during cell culture (data not shown). TGF-β-stimulated hepatocytes significantly enhance their CTGF expression (Fig. 1C, 1D) in a time dependent manner (Fig. 2). Moreover, dexamethasone promotes CTGF expression in comparison to untreated hepatocytes and a combined incubation with TGF-β leads to a significant "super induction" of CTGF.

### Direct secretion of PAI-1 and delayed secretion of CTGF by hepatocytes

PAI-1 and CTGF contribute for ECM production and deposition. For this purpose they were secreted. Hepatocytes were treated with TGF-β or TGF-β and dexamethasone and incubated for 1, 2, and 4 hours, respectively. The time course of stimulated hepatocytes showed an increasing amount of PAI-1 in cell lysate and medium after TGF-β incubation or a TGF-β-dexamethasone cotreatment (Fig. 2). We observed that the PAI-1 export into medium exceed the PAI-1 concentration of the cell lysate in the untreated control (Fig. 2, lane 7). A comparison of the untreated cells depicted a continuous increase of PAI-1 in medium samples of hepatocytes. In contrast to PAI-1, the smaller CTGF protein was not observed in notably amounts in medium samples within a time-frame from 1 to 4 hours, although a time-dependent increase of CTGF protein in lysates was detected. But after 4 hours the band intensity of CTGF increased, which was facilitated in those samples treated with TGF-β (Fig. 1C – 6 h). Additional experiments showed that only weak CTGF bands were present in medium samples after stimulations with various dexamethasone concentrations (data not shown), indicating that only TGF-β treated samples triggers CTGF secretion in hepatocytes.

Life cell observations showed that the combination of TGF-β and dexamethasone in the used concentrations did not change cell phenotype. A lactate dehydrogenase determination of co-treated, single treated or untreated hepatocytes averages from 28–33 LDH U/l (37°C), indicating that no toxic procedures were initiated, which affect cell integrity.

### GR reduces TGF-β signalling and vice versa TGF-β inhibits GR signalling in Hep-G2 cells

**Figure 3:**
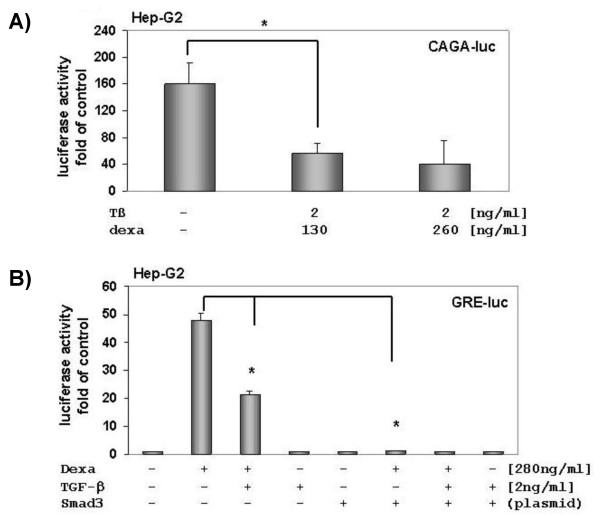
**Effect of dexamethasone on a TGF-β-Smad 3-sensitive reporter construct [(CAGA)_9_- MLP-luc]**. (A) Dexamethasone inhibits TGF-β-Smad 3-signalling in Hep-G2. Hep-G2 was transfected with (CAGA)_9_- MLP-luc and stimulated with 1 or 2 ng of TGF-β1. Four hours after incubation with various dexamethasone concentrations, cells were lysed and luciferase reporter gene activity was measured. Results are presented as light units relative to untreated cells. The presented data is the mean ± SD of three independent experiments, each performed in duplicates. (B) TGF-β decreases GR signalling. Hep-G2 cells were co-transfected with GRE4E1b-luc and Smad 3 plasmid. TGF-β decreases and over-expressed Smad 3 abrogates GR signalling. Luciferase activities were measured using the Victor 1420 Multilabel Counter Luminometer (Wallac, Germany).

In previous studies we found that a TGF-β-Smad 3-sensitive reporter construct could be repressed by corticosteroids in activated HSC [21]. Additionally, we observed that TGF-β induced PAI-1 mRNA expression is inhibited by dexamethasone (data not shown). To check the interaction between dexamethasone and TGF-β-Smad 3 signalling we first transfected Hep-G2 with a (CAGA)_9_-MLP-luc plasmid, containing nine Smad-binding elements from PAI-1 promoter. Our data shows that dexamethasone suppresses TGF-β-induced luciferase signal significantly (Fig. 3A). *Vice versa *transfection studies with a glucocorticoid-sensitive reporter construct showed that GR transactivation was clearly inhibited by TGF-β and ectopic Smad 3 (Fig. 3B).

### Effects of Erk and JNK inhibitor on CTGF and PAI-1 expression in hepatocytes

In addition to the Smads, TGF-β is able to signal via Ras-Mek-Erk pathway and activate the extra cellular signal-regulated kinases (Erk1 = p44 and Erk2 = p42) and the Jun-N-terminal kinase (JNK) [23,24]. This settles the question of whether TGF-β or dexamethasone-induced CTGF or PAI-1 expression were connected with the Erk- or JNK-pathway.

**Figure 4:**
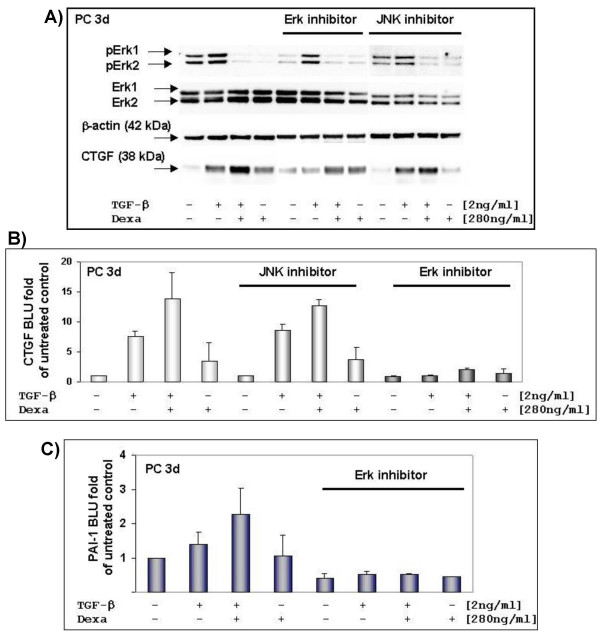
**Effects of JNK inhibitor II and Erk inhibitor on TGF-β and dexamethasone induced CTGF expression in hepatocytes**. (A) Three-days old hepatocytes were serum-starved before stimulation. Cells were treated with or without inhibitor and then stimulated for three hours with TGF-β and dexamethasone as indicated and thereafter immediately lysed. Cell lysates have been subjected to electrophoresis and Western blot. Corresponding expression of β-actin is shown as loading control. (B) Densitometrically evaluation of independent cell preparations showed that CTGF expression was not changed in the presence of JNK inhibitor (n = 2; light-grey). In comparison to untreated cells (n = 3; white bars) the addition of Erk inhibitor clearly inhibited TGF-β/dexamethasone induced CTGF expression (n = 2; dark-grey). BLU (Boehringer light units). (C) Just as well, densitometric analysis of PAI-1 demonstrated that the Erk inhibitor repressed TGF-β and dexamethasone induced expression. (Native – n = 4; with Erk inhibitor – n = 2).

Our data demonstrate that TGF-β and TGF-β dexamethasone-induced CTGF expression was not affected by inhibition of the c-Jun phosphorylation (Fig. 4A). We observed that dexamethasone strongly dephosphorylates Erk1 and Erk2, which could be antagonized by mifepristone (data not shown), whereas TGF-β induced their phosphorylation. The addition of an Erk inhibitor, a 13 amino-acid peptide, which selectively prevents interaction between Mek and Erk and thereby specifically inhibits downstream Erk activation, reduced TGF-β promoted CTGF and PAI-1 expression (Fig. 4B, 4C). The dexamethasone induced CTGF expression was not affected by the Erk inhibitor (Fig. 4A). We ascertain that Erk2 expression level is higher than Erk1 in three-day old hepatocytes. In contrast to the Erk inhibitor, an inhibition of JNK-activation had also no effect on TGF-β-induced PAI-1 expression (data not shown).

## Discussion

It is well established that PAI-1 and CTGF are involved in the development of liver fibrosis and that they are both target genes of TGF-β as well as dexamethasone (see synopsis of potential response elements in Table 1). Hence, this poses the question how their expression and availability is regulated in hepatocytes.

**Table 1:** Synopsis of the predicted TGF-β and GR response elements in PAI-1 and CTGF promoter sequences

**Gene**	**Promoter motifs**	**Pathway**	**Promoter sequence**	**Ref.**
hCTGF	GTGTCAAGGGGTCAGGAT	TGF-β inducible	-162 to 128	[14]
hCTGF	GAGGAATGCGAGGAATG	TGF-β-Smad3/4	-805 to +17	[15]
hCTGF	**-**	GR	-292 to +22	[25]
mCTGF	No direct binding, interaction with other TF (STAT3?)	GR	-897 to -628 (mouse strain specific)	[19]
hPAI-1	AG(C/A)CAGACA	TGF-β-Smad3/4	-730, -580, -280	[26]
hPAI-1	AG(C/A)CAGACA	TGF-β-Smad3/4	-732 to -721	[27]
PAI-1	MLP(CAGA)_9_-luc	TGF-β-Smad3/4	Reporter plasmid	[28]
hPAI-1	-	GR	-800, -549, -100, +75,	[17]
rPAI-1	AGCACACACTGTTCT	GR	-1212 to -1196	[29]

Besides the transcriptional activation of gene expression through potentially response elements of single transcription factors, and apart from a cell-type specific composition of those transcription factors, a superior regulation is conceivable, *e.g*. a diversity in methylation sites or the condensation status of the chromatin. GTCTAGAC: TGF-β-Smad3/4 consensus sequence; GGTACANNNTGTTCT: GR consensus sequence; r: rat; h: human; m: mouse.

Multiple pathways are involved in transcriptional regulation of PAI-1 e.g. TGF-β-Smad and TGF-β-MAPK, but not all mechanisms and interactions are completely understood [17,23-30]. We showed that TGF-β induces PAI-1 expression in primary hepatocytes, which is consistent with previous findings in HSC and hepatocytes [31,32]. Although dexamethasone antagonized a TGF-β sensitive luciferase reporter consisting of nine CAGA-elements from PAI-1 promoter, it significantly enhanced TGF-β-induced PAI-1 in hepatocytes. Similar to our findings in hepatocytes, Ma and coworkers found an increase of TGF-β-induced PAI-1 expression through dexamethasone in HTR-8 SV neo cells [33]. They suggested that dexamethasone enhancement on TGF-β-mediated PAI-1 expression is not subjected to a direct transcriptional mechanism, but rather through an additional protein. A successful TGF-β-induced PAI-1 expression seems to be a positive cooperation between the Smads and activator protein-1 (AP-1) [30]. The AP-1 activity is supposed to be a result of a directly TGF-β-induced MAPK signalling. Depending on the composition of AP-1 (Jun-Fos, Jun-Jun or Jun-ATF2) a positive or negative interaction with GR is possible [34,35]. But we observed that an inhibition of c-Jun phosphorylation did not affect PAI-1 expression in hepatocytes. The inhibition of Mek-Erk interaction clearly suppressed TGF-induced PAI-1, which points to cooperative signalling between TGF-β and Erk-pathway for regulating PAI-1 expression. In agreement with other groups [31,36], we observed a low PAI-1 level in primary hepatocytes, but when PAI-1 was induced it was directly secreted.

Of particular concern is the fact that a costimulation with both, TGF-β and dexamethasone, also strongly induce the profibrogenic CTGF protein expression in hepatocytes. In fibroblasts TGF-β-induced CTGF is enhanced by Ras/Mek/Erk signalling and suppressed by JNK signalling and it seems that Ras/Mek/Erk did not affect the Smad pathway [15]. In agreement with Leask et al. [15] we found that TGF-β induction of CTGF is dependent on Erk-activity in hepatocytes. In contrast to the fibroblasts an inhibition of c-Jun phosphorylation through JNK did not change TGF-β induced CTGF expression.

**Figure 5:**
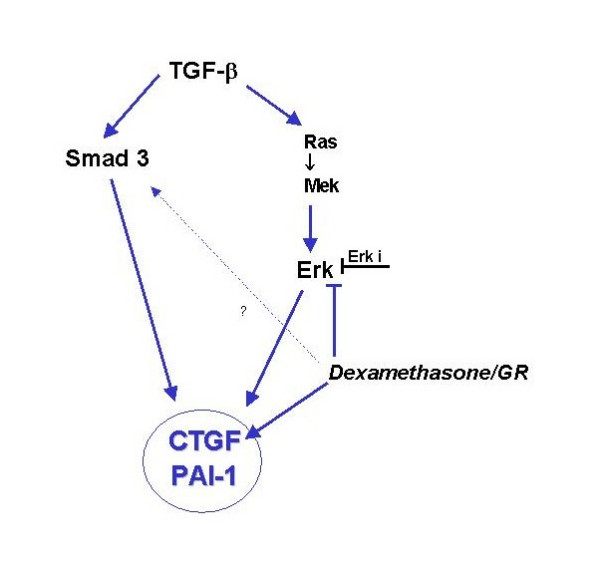
**Diagram of the proposed role of Erk-pathway in the control of CTGF and PAI-1 expression**. CTGF and PAI-1 seems to be regulated by two TGF-β-induced pathways: TGF-β-Smad 3 and TGF-β-Ras-Mek-Erk pathway. Additionally, dexamethasone clearly induced CTGF expression but it did not affect PAI-1 expression in hepatocytes. [TGF-β] *transforming growth factor beta*, [Smad 3] *mothers against DPP homolog 3*, transcription factor, [Ras] *rat sarcoma*, proto-oncogen, [Mek] *MAP-Erk-Kinasen*; [Erk] *Extra-cellular-signal regulated Kinase*; [dexamethasone] glucocorticoid, transcription factor; [JNK] *c-Jun N-terminale Kinase*; [Erk i] Erk inhibitor binds selectively to Erk2 and prevent its interaction with Mek.

How can we explain the synergistic effect of dexamethasone and TGF-β on PAI-1 and CTGF expression in hepatocytes? On the one hand, glucocorticoid bound GR inhibits a TGF-β-sensitive reporter signal and, *vice versa*, TGF-β and/or ectopic Smad 3 expression decreased a glucocorticoid-sensitive luciferase reporter in Hep-G2. These results indicate an antagonizing interaction between the TGF-β-Smad and the glucocorticoid pathway, *e.g*. via the proposed GR-Smad 3 protein-protein interaction [37,38]. However, the dexamethasone enhancement of TGF-β up-regulated CTGF could be antagonized by mifepristone, which argue for a GR-dependent synergistic effect in primary hepatocytes. On the other hand, it is conceivable that cell type-specific co-regulators directed the hormonal response by preventing a GR-Smad interaction and facilitating a subsequent DNA binding. We suppose that the TGF-β mediated induction of CTGF or PAI-1 expression needs the Smad pathway as well as the Ras-Mek-Erk-pathway (Fig. 5). In fact, our data demonstrate that a simultaneous stimulation with TGF-β and dexamethasone leads to a "super induction" of PAI-1 and CTGF in hepatocytes, which argue for synergistic transcriptional gene activation and against a direct GR-Smad 3 crosstalk. Additionally, our results raise the intriguing possibility that CTGF requires a factor, which is permissive for a real-time secretion by the cells. Overall, our results provide new evidence that in hepatocytes CTGF is regulated via the TGF-β-Smad 3- and the TGF-β-Ras-Mek-Erk- as well as the GR-pathway.

## Conclusion

In summary, our results clearly showed a synergistic effect of dexamethasone on TGF-β-induced PAI-1 and CTGF expression in primary hepatocytes, which can be selectively inhibited by supressing the Mek-Erk activity. The differential secretion of PAI-1 and CTGF is suggested to be relevant in organized extracellular matrix deposition and thus in liver cell interaction.

## Materials and methods

### Materials

Dexamethasone was obtained from Sigma (Taufkirchen, Germany). Recombinant human TGF-β1 was obtained from R&D systems (Minneapolis, MN). Erk activator inhibitor protein (328000) and JNK inhibitor II (420119) were purchased from Calbiochem.

### Cell isolation and culture

Hepatocytes were isolated from Sprague-Dawley rats (250 g) as described by Seglen [39], seeded with a density of 5 × 10^6 ^cells/cm^2 ^and cultured in Dulbecco's modified Eagle's medium (DMEM, Bio-Whittaker Europe, Verviers, Belgium), supplemented with 4 mmol/l L-glutamine, 10 % FCS, and penicillin (100 IU/ml)/streptomycin (100 μg/ml). All cultures were maintained at 37°C, 5 % CO_2_/95 % air in a humidified atmosphere. HepG2 cells [40] were cultured in supplemented DMEM described above. Hepatocytes were treated with cytokines and/or hormones under serum free conditions. The inhibitors (Erk inh. 0.2 μM, JNK inh. 10 μM) were pre-incubated about 10 to 30 minutes before dexamethasone or TGF-β stimulation.

### Lactate dehydrogenase assay (LDH)

Lactate dehydrogenase activity was assayed using the IFCC method (International Federation of Clinical Chemistry and Laboratory Medicine), at 37°C, in the media of hepatocytes treated with TGF-β and dexamethasone as indicated. The culture media were harvested and LDH was measured with Roche P800 modular (Roche Diagnostics GmbH, Germany).

### Western blotting

Freshly isolated hepatocytes were seeded in 90 mm^2 ^culture dishes at a density of 5.0 × 10^6 ^cells. Directly after serum starvation, cells were exposed for TGF-β, dexamethasone or both as indicated. Thereafter, cells were harvested and the protein concentration was determined by MicroBC assay protein quantification kit (Interchim, Montluçon, France). Equal amounts of protein (40 μg) were separated in a 4–12 % Bis-Tris NuPAGE gel (Invitrogen) and transferred to a 0.45 μm nitrocellulose membrane (AppliChem, Darmstadt, Germany). Blots were washed and blocked with 5 % milk/TBST over night at 4°C. Primary antibodies: PAI-1 (Abcam, Cambridge, UK), CTGF (Santa Cruz Biotechnology, Santa Cruz, CA, USA), β-actin (Sigma); Phospho-p44/42 MAP kinase (Erk1/Erk2; 9101s) and p44/42 MAP kinase (9102) were purchased from Cell Signalling. The phospho-p44/42 MAP kinase antibody detects endogenous level of phosphorylated Thr202/Tyr204 of Erk1 and Thr183/Tyr185 of rat Erk2. The first antibody was incubated 2 h at RT and the second antibody (HRP conjugated; Santa Cruz) was incubated for 1 h at RT, respectively, both diluted in 2.5 % milk/TBST. Blots were developed with SuperSignal West Dura (Pierce, Bonn, Germany). Densitometric analysis has been carried out with Lumi Imager (Boehringer Mannheim; software version 3.0). Significance of differences was established by the paired Student's *t*-test.

### Transfection and luciferase assay

Hep-G2 cells were seeded in 12- or 24-well plates at a density of 3.0 - 2.5 × 10^4 ^cells/well. To measure TGF-β signalling the TGF-β- sensitive reporter construct, (CAGA)_9 _-MLP-luc [26] was transiently transfected by using FuGENE 6 (Roche). GR-signalling was measured by using the GRE4E1b-luc reporter, kindly provided by Gelehrter (University of Michigan, USA; [37]). Smad 3 plasmid was kindly provided by S. Dooley (University Mannheim, Germany), respectively.

## Competing interests

The author(s) declare that they have no competing interests.

## Authors' contributions

KK and NC performed most of the experiments and provide assitance for the preparation of the manuscript. LW and AMG participate in the design of the study and prepared the manuscript. All authors have read and approved the content of the manuscript.
